# Virus-Infected Plants Altered the Host Selection of *Encarsia formosa*, a Parasitoid of Whiteflies

**DOI:** 10.3389/fphys.2017.00937

**Published:** 2017-11-22

**Authors:** Xin Liu, Gong Chen, Youjun Zhang, Wen Xie, Qingjun Wu, Shaoli Wang

**Affiliations:** ^1^Department of Plant Protection, Institute of Vegetables and Flowers, Chinese Academy of Agricultural Sciences, Beijing, China; ^2^College of Plant Protection, Hunan Agricultural University, Changsha, China

**Keywords:** *Encarsia formosa*, *Bemisia tabaci*, tomato yellow leaf curl virus, host selection, plant volatiles

## Abstract

The sweetpotato whitefly *Bemisia tabaci* (Gennadius) is one of the most invasive pest species worldwide. Q and B biotypes are the two most devastating species within the *B. tabaci* complex. *Bemisia tabaci* can vector hundreds of plant viruses that seriously threaten crop production. Endoparasitoid, *Encarsia formosa* Gahan, is widely used to control whiteflies, however, little is known about the effects of virus-infected plants on *E. formosa* parasitism of *B. tabaci*. Here, we reported that tomato, which was infected with *Tomato Yellow Leaf Curl Virus* (TYLCV), altered the host selection of *E. formosa* between *B. tabaci* Q and B biotypes. On healthy tomato plants, parasitism and host selection by *E. formosa* did not differ between the 3rd-instar nymphs of *B. tabaci* Q and B biotypes. On TYLCV-infected tomato plants, however, *B. tabaci* Q biotype were significantly more attractive to *E. formosa* than B biotype. When TYLCV-infected tomato plants were infested with *B. tabaci* Q or B biotype, volatile profiles differed quantitatively but not qualitatively. Olfactometer assays suggested that the preference of *E. formosa* to Q over B biotype was associated with an elevated level of β-Myrcene, β-Ocimene, β-Caryophyllene, and α-Humulene from TYLCV-infected tomato plants.

## Introduction

Parasitoid wasps, during foraging, may encounter various and multiple situations or factors, such as insect species and these insect-borne pathogens; this would make the parasitoids foraging more complicated and difficult (Ponzio et al., 2013, 2016a; Li et al., 2014; Martini et al., 2014; Mauck et al., 2015). Hence, finding a reliable and efficient way to locate their suitable host is important for their propagation. Numerous evidences have indicated that the pathogen, herbivory or oviposition-induced plant volatiles are important infochemicals and reliable indicators to many parasitoid species for seeking and locating their hosts (McCormick et al., 2012; Li et al., 2014; Martini et al., 2014; Mauck et al., 2015; Ponzio et al., 2016a,b). These chemical volatiles thus have an important role for the host searching, location and discrimination behaviors of carnivores (McCormick et al., 2012; Li et al., 2014; Ponzio et al., 2016a,b).

The sweetpotato whitefly, *Bemisia tabaci* is an important insect pest on many vegetables and other agricultural crops. The Q and B biotypes are two most invasive and destructive cryptic species of *B. tabaci* in China (De Barro et al., 2011; Pan et al., 2012; Zheng et al., 2017). Serious damage by *B. tabaci* results not only from its removal of plant sap and secretion of honeydew (which causes sooty mold) but also from its transmission of various begomoviruses (De Barro et al., 2011). The only known vector of *Tomato yellow leaf curl virus* (TYLCV), which is a typical begomovirus, is *B. tabaci*, and *B. tabaci* transmits TYLCV in a persistent manner (Brown and Czosnek, 2002; Jones, 2003). A recent study found that TYLCV transmission efficiency is higher for the Q biotype than for the B biotype (Ning et al., 2015). Although the Q biotype has replaced the B biotype in many areas of China over the past decades (Pan et al., 2011), the two biotypes continue to coexist in some locations (Chu et al., 2010a,b; Teng et al., 2010; Pan et al., 2012; Zheng et al., 2017).

*Encarsia formosa* Gahan is a cosmopolitan and commercially important parasitoid of *B. tabaci* and other whiteflies, and has been widely used to reduce whitefly damage to crops (Gerling et al., 2001; Grille et al., 2012; Liu et al., 2015, 2016). Parasitism of *B. tabaci* by *E. formosa* is affected by the developmental stage of Q and B biotypes (Liu et al., 2016) and may also be affected by infection of host plants by TYLCV (Liu et al., 2014). In the latter case, parasitism of Q biotype nymphs was higher on TYLCV-infected tomato plants than on TYLCV-free plants, but parasitism of B biotype nymphs did not significantly differ on TYLCV-infected vs. TYLCV-free tomato plants (Pan et al., 2013, 2014; Liu et al., 2014). These studies suggested that the selection of *B. tabaci* biotype Q and B as hosts by *E. formosa* may differ and may be affected by TYLCV infection of plants. Additional research is needed to determine how parasitism and host selection by *E. formosa* differs between the two important biotypes of *B. tabaci* and how such selection and parasitism is affected by TYLCV infection of host tomato plants.

The first objective of the present study was to further describe how host selection by *E. formosa* differs between *B. tabaci* Q and B biotypes and how host selection is affected by TYLCV infection of tomato plants. A second objective was to determine potential mechanisms underlying the effects of biotype and TYLCV infection of tomato on such host selection.

## Materials and methods

### Plant and insect materials

*Lycopersicon esculentum* (variety “No. 9 Zhongza”) tomato plants were grown in plastic pots (7 cm diameter) containing a mixture of peat moss, vermiculite, organic fertilizer, and perlite in a 10:10:10:1 ratio by volume (Shi et al., 2013). Tomato plants with three to four expanded leaves were inoculated with a TYLCV infectious clone kindly provided by Professor Xueping Zhou (Institute of Plant Protection, Chinese Academy of Agricultural Sciences, Beijing, China); infection by TYLCV was confirmed as previously described (Ghanim et al., 2007; Shi et al., 2013; Liu et al., 2014). TYLCV-free plants were not inoculated with the virus. Plants with six to eight fully expanded leaves were used for the assays and experiments described in the following sections.

Laboratory colonies of *B. tabaci* biotype Q and B, whose identities was periodically confirmed based on the cleavage amplified polymorphic sequence (CAPS) and mitochondrial cytochrome oxidase I genes (*mtCOI*) (Chu et al., 2010a), were separately maintained on virus-free tomato plants in insect-proof cages in a greenhouse. *Encarsia formosa* was provided by the Beneficial Insects Research Center, Shandong Academy of Agricultural Sciences in China; the parasitoid had been reared on nymphs of *Trialeurodes vaporariorum* (Westwood) for >10 years.

### *Encarsia formosa*'s parasitism on *B. tabaci* Q and B biotypes under *in Vitro* and semi-natural conditions

Selection and oviposition of *E. formosa* on HQ vs. HB or VQ vs. VB were compared under *in vitro* conditions in the laboratory. HQ and HB refer to virus-free plants infested with 3rd-instar nymphs of *B. tabaci* Q or B. VQ and VB refer to virus-infected plants infested with 3rd-instar nymphs of *B. tabaci* Q or B. TYLCV-infected or TYLCV-free tomato leaves were infested with 30 3rd-instar nymphs of *B. tabaci* biotype Q or B, and *in vitro* parasitism was assessed as previously described (Liu et al., 2016). In brief, the leaves infested by 3rd-instar nymphs of *B. tabaci* biotype Q or B were detached from the TYLCV-free or TYLCV-infected plants. The leaf petioles were wrapped in cotton wool saturated with distilled water. Each treatment pair (HQ vs. HB and VQ vs. VB) was placed in plastic Petri dishes (diameter 8.5 cm, height 1.5 cm, two leaves per dish). A filter paper disk saturated with distilled water was also placed beneath each leaf. Newly emerged *E. formosa* females (1 day old; wasps: nymphs = 1: 30) were subsequently released in the center of each Petri dishes between the two leaves and were carefully removed after 24 h. About 8–10 days later, the number of nymphs parasitized by wasps was recorded. Each comparison was carried out for 19 replicates.

To confirm the results obtained from the *in vitro* experiment, a greenhouse experiment that simulated field conditions was carried out as described by Zhang et al. (2013b). *Bemisia tabaci* biotype Q or B adults were placed in clip-cages on the leaves of TYLCV-infected or TYLCV-free plants (four clip-cages per plant, 20 adults per clip-cage) and allowed to oviposit; after 24 h, all of the adults were removed. About 20 days later, when most eggs had hatched and developed into 3rd-instar nymphs, one plant of each treatment pair (HQ vs. HB and VQ vs.VB) was placed in a ventilated cage (88 × 60 × 79 cm); the two plants in each cage were 50 cm apart. Newly emerged *E. formosa* females (1 day old; wasps: nymphs = 1: 30) were subsequently released in the center of each cage between the two plants. After 24 h, we carefully checked the tomato leaves and removed the parasitoids by using a small suction trap. The viruliferous and TYLCV-free tomato plants carrying nymphs were separately maintained in a climate chamber at 26 ± 1°C with 70 ± 5% relative humidity and a 16 h/8 h light/dark photoperiod. About 8–10 days later, the number of nymphs parasitized by wasps was recorded. Each comparison was replicated four times, and replications were conducted on different days to account for day-to-day variation (Zhang et al., 2013b).

### Olfactometer assays

A Y-tube olfactometer was used to assess the behavioral responses of *E. formosa* to the following pairs of odor sources: TYLCV-free tomato plants infested with 3rd-instar nymphs of *B. tabaci* biotype Q (HQ) or B (HB); TYLCV-infected tomato plants infested with 3rd-instar nymphs of *B. tabaci* biotype Q (VQ) or B (VB). The following combinations were also carried out to verify the absence of differences within each treatment: TYLCV-infected tomato plants without *B. tabaci* infestation (V) vs. V; TYLCV-free tomato plants without *B. tabaci* infestation (H) vs. H; VQ vs. VQ; VB vs.VB; HQ vs. HQ; and HB vs. HB. In all cases involving infestation, plants were infested with approximately 20 nymphs per leaf.

The olfactometer assays were carried out as previously described (Zhang et al., 2013b). In brief, an adult female of *E. formosa* (1.0–2.0 days old) was released at the base of the Y-tube and then observed continuously for 5 min. Each female was tested only once. The selection of the two odor sources was recorded when the female had moved into one arm of the Y-tube for at least one-third of the arm's length and remained for at least 15 s; a female that walked to the far end of the arm was also recorded as a selection. If the female did not choose within 5 min, “no choice” was recorded. After five females were tested, odor sources were interchanged to avoid any influence of asymmetries in the set-up system. The Y-tube was replaced with a new one after it had been used to test 10 wasps. To avoid the influence of residual odor, each used tube was rinsed with 75% alcohol and kept in an oven at 180°C overnight. Each comparison was replicated three times, with 20 females per replicate.

### Collection and analysis of plant volatiles

Volatiles emitted from TYLCV-infected or TYLCV-free tomato plants infested by nymphs of *B. tabaci* biotype Q or B were collected and analyzed as described previously (Wei et al., 2007; Chen et al., 2017). The potted plants, which were carefully wrapped in aluminum foil with only the above-ground green part exposed, were placed in collection bags (one plant per bag) with a gas inlet and a gas outlet. Purified and humidified air at 300 mL min^−1^ was pumped into each bag through the inlet. A glass tube filled with 0.1 g of PoraPak Q (80/100-mesh; Waters, USA) was used to trap plant volatiles for 4 h at the outlet under continuous high-intensity sodium-halide light. Volatiles were collected from four replicate plants per treatment.

The trapped volatile samples were eluted from the PoraPak Q with 800 μL of high-performance liquid chromatography (HPLC)-grade methylene chloride (Tedia Company, Fairfield, Ohio, USA); an internal standard (10 μL of 20 ng/μL of n-octane) was added to each sample for quantification of relative compound amounts. A 1-μL sample of the solution was subjected to gas chromatography–mass spectrometry (GC-MS); the gas chromatograph (GC-2010 Shimadzu, Japan) was equipped with an Agilent Technologies capillary column DB-5MS (30 m × 0.25 mm ID × 0.25 μm film thickness). The temperature program was as follows: the initial oven temperature was kept at 40°C for 4 min and was then increased to 200°C at a programmed rate of 5°C min^−1^, followed by a rate at 20°C min^−1^ to 280°C. The injector temperature was maintained at 250°C with a constant flow rate of 1.0 mL min^−1^. Compounds were identified by comparison of retention times and mass spectra (NIST database and synthetic standards). The peak area of the volatile expressed as a percentage of the peak area of the internal standard was used to analysis the change of volatiles (Chen et al., 2017).

### Attraction verification of *E. formosa* to synthetic volatiles

A Y-tube olfactometer was used to assess the attractions of candidate volatile compounds to *E. formosa* females as previously described (Zhang et al., 2013b). The candidate compounds (β-Myrcene, β-Ocimene, β-Caryophyllene, α-Humulene, and β-Elemene) were selected based on data obtained with TYLCV-infected tomato plants infested with *B. tabaci* nymphs (see Results). Nine pairs of treatments were compared: Paraffin oil (CK) vs. CK, β-Myrcene (4.350 ng/μl) vs. CK, β-Ocimene (3.525 ng/μl) vs. CK, β-Caryophyllene (3.350 ng/μl) vs. CK, α-Humulene (1.625 ng/μl) vs. CK, β-Elemene (0.675 ng/μl) vs. CK, and mixture of monoterpenes (β-Myrcene and β-Ocimene) vs. CK, mixture of sequiterpenes (β-Caryophyllene, α-Humulene, and β-Elemene) vs. CK, mixture of total candidate compounds vs. CK. As indicated, paraffin oil (CK) was used as the solvent. Two streams of purified and humidified air at 300 ml min^−1^ were separately passed through two glass containers containing the test volatile in paraffin oil or containing only paraffin oil as a control and into the olfactometer arms (Chen et al., 2017). The experimental method and data collection were the same as described earlier for the olfactometer assays.

### Statistical analysis

Chi-squared tests were used to analyze the Y-tube olfactometer assays (significance level: *P* < 0.05) (Martini et al., 2014; Mauck et al., 2015). Parasitoids that did not make a choice were excluded from the analysis. Independent-sample *t*-tests were used to compare the percentage of *B. tabaci* nymphs parasitized by *E. formosa* in each treatment pair and the quantities of volatile compounds collected from TYLCV-infected or TYLCV-free tomato plants infested with nymphs of *B. tabaci* biotype Q or B. All proportional data were first checked for normality and were transformed when necessary to meet the assumption of normal distribution before analysis. SPSS version 19.0 (SPSS Inc., Chicago, IL, USA) was used for all statistical analysis (Liu et al., 2016).

## Results

### *Encarsia formosa*' s parasitism on *B. tabaci* Q and B biotypes under laboratory and semi-natural conditions

**Figure 1:**
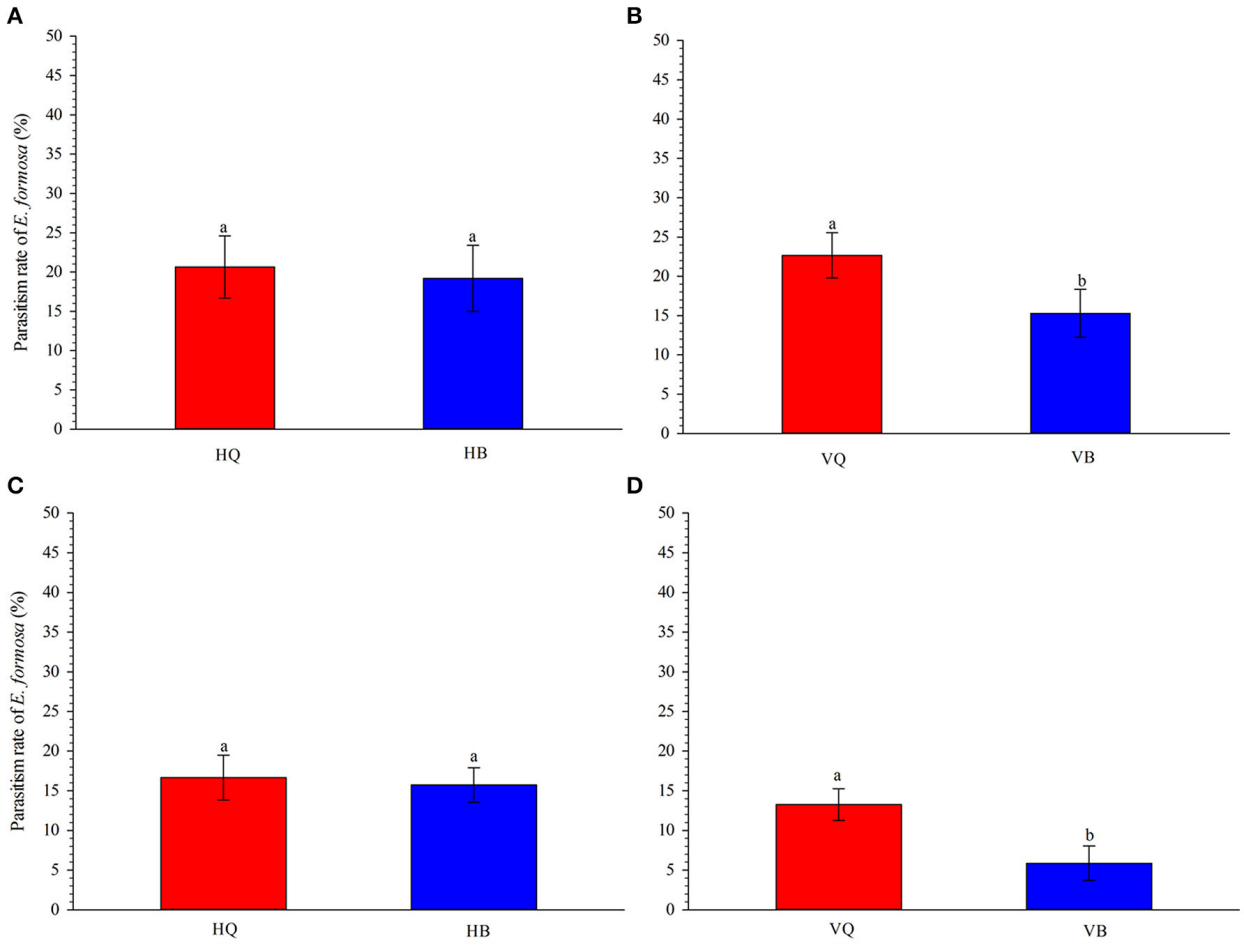
Parasitism of 3rd-instar nymphs of *Bemisia tabaci* biotype Q vs. B by *E. formosa* on TYLCV-free **(A)** and TYLCV-infected **(B)** tomato plants under *in vitro* conditions and on TYLCV-free **(C)** and TYLCV-infected **(D)** plants under semi-natural conditions. HQ and HB refer to TYLCV-free plants infested with biotype Q or B. VQ and VB refer to virus-infected plants infested with biotype Q or B. Values are means ± SE. In each panel, means with different letters are significantly different according to the Tukey test at *P* < 0.05.

On tomato leaves in the laboratory, parasitism of 3rd-instar nymphs of *B. tabaci* by *E. formosa* females did not differ between biotype Q and B on TYLCV-free tomato plants (*P* = 0.834, Figure 1A). On TYLCV-infected tomato plants, however, parasitism was significantly higher for biotype Q than B (*P* = 0.031, Figure 1B).

The results of the semi-natural experiment showed the same trends as that in the laboratory experiment. The *E. formosa* did not show a preference for Q or B biotype on TYLCV-free tomato plants (*P* = 0.797, Figure 1C) but preferred biotype Q to biotype B on TYLCV-infected tomato plants (*P* = 0.027, Figure 1D).

### Olfactometer assays

**Figure 2:**
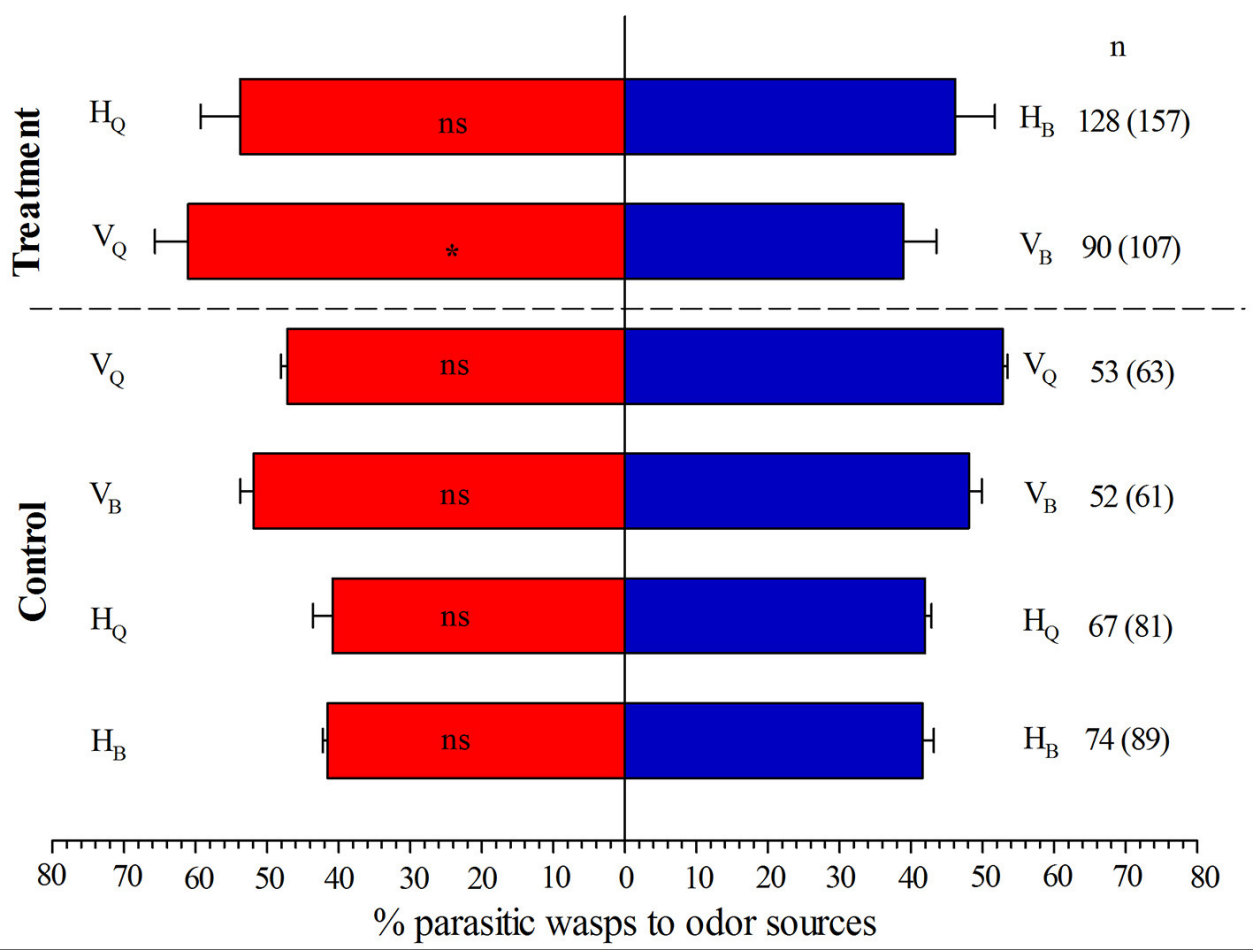
Responses of *Encarsia formosa* to odor sources in a Y-tube olfactometer. Bars (means + SE) indicate the percentages of wasps choosing either of the odor sources. HQ /HB, TYLCV-free tomato plants infested with 3rd-instar nymphs of *B. tabaci* biotype Q/B; VQ/VB, TYLCV-infected tomato plants infested with 3rd-instar nymphs of *B. tabaci* biotype Q/B. The numbers to the right of bars indicate the number of wasps making a choice, and the total number of wasps used in the assay is indicated in parentheses. Asterisks indicate significant differences between the treatment pairs (χ^2^ test; ^*^P < 0.05; ns, not significant).

When tomato plants were not infected with TYLCV in Y-tube olfactometer assays, the naive *E. formosa* females showed no significant preference for plants infested with 3rd-instar nymphs of biotype Q vs. biotype B of *B. tabaci* (*P* = 0.424, Figure 2). When tomato plants were infected by TYLCV, however, *E. formosa* significantly preferred tomato plants infested with biotype Q rather than biotype B (*P* = 0.046, Figure 2).

### Analysis of plant volatiles

According to the analysis of the plant headspace volatiles by gas chromatography-mass spectrometry (GC-MS), all treatments produced the same 32 compounds, including monoterpenes, sequiterpenes, aldehydes, alcohols, and esters (Table 1), and differences between treatments were quantitative rather than qualitative. Quantitative analysis revealed that emissions of the monoterpenes including β-Myrcene and β-Ocimene, and the sesquiterpenes including β-Caryophyllene, α-Humulene, and β-Elemene were higher from TYLCV-infected plants infested with 3rd-instar nymphs of *B. tabaci* Q than with nymphs of *B. tabaci* B. The amounts of these compounds emitted from TYLCV-free plants did not significantly differ when the plants were infested with 3rd-instar nymphs of *B. tabaci* Q vs. B (Table 1).

**Table 1:** The peak area ratios of volatile components released from tomato plants infested by the 3rd-instar nymphs of *B. tabaci*.

**Compound**	**Retention time (min)**	**H_Q_ (mean ± SE)**	**H_B_ (mean ± SE)**	***P***	**V_Q_ (mean ± SE)**	**V_B_ (mean ± SE)**	***P***
Methyl-Benzene	5.046	2.67 ± 0.16a	2.11 ± 0.24a	0.099	2.18 ± 0.11a	1.84 ± 0.16a	0.145
Hexanal	6.104	0.21 ± 0.33a	0.29 ± 0.05a	0.183	0.20 ± 0.03a	0.14 ± 0.02a	0.101
1-Octene	7.322	0.37 ± 0.02a	0.37 ± 0.01a	0.721	0.26 ± 0.03a	0.22 ± 0.02a	0.260
Mthyl-Benzene	7.994	0.50 ± 0.07a	0.47 ± 0.08a	0.755	0.28 ± 0.03a	0.25 ± 0.02a	0.369
O-Xylene	9.08	0.45 ± 0.06a	0.43 ± 0.07a	0.836	0.25 ± 0.02a	0.22 ± 0.01a	0.203
α-Pinene	10.53	9.96 ± 0.38a	9.75 ± 1.44a	0.733	12.35 ± 2.77a	9.31 ± 0.76a	0.238
β-Cymene	11.875	11.61 ± 0.54a	10.13 ± 1.17a	0.235	10.26 ± 1.37a	9.47 ± 1.49a	0.703
β-Pinene	12.081	0.43 ± 0.03a	0.33 ± 0.06a	0.109	0.52 ± 0.10a	0.32 ± 0.04a	0.086
β**-Myrcene**	12.545	1.15 ± 0.11a	0.87 ± 0.10a	0.087	1.79 ± 0.15a	1.10 ± 0.05b	0.003
(+)-4-Carene	12.84	50.19 ± 2.89a	43.17 ± 6.76a	0.314	44.25 ± 9.16a	41.45 ± 3.49a	0.072
l-Phellandrene	13.088	16.98 ± 1.22a	14.00 ± 2.41a	0.259	11.61 ± 0.83a	14.75 ± 1.30a	0.069
(+)-2-Carene	13.453	3.36 ± 0.25a	2.87 ± 0.55a	0.392	2.79 ± 0.49a	3.10 ± 0.33a	0.607
A	13.719	1.34 ± 0.07a	1.11 ± 0.13a	0.105	1.54 ± 0.23a	1.02 ± 0.10a	0.080
β-Phellandrene	13.926	210.95 ± 15.22a	187.68 ± 28.69a	0.457	189.09 ± 14.63a	158.75 ± 18.55a	0.228
β**-Ocimene**	14.469	0.20 ± 0.02a	0.19 ± 0.03a	0.707	0.21 ± 0.05a	0.098 ± 0.01b	0.037
B	14.843	0.73 ± 0.06a	0.58 ± 0.11a	0.237	0.47 ± 0.04a	0.55 ± 0.03a	0.147
α-Terpinolene	15.731	0.74 ± 0.07a	0.65 ± 0.14a	0.517	0.82 ± 0.16a	0.49 ± 0.07a	0.083
Tridecane	16.227	0.33 ± 0.01a	0.31 ± 0.02a	0.393	0.15 ± 0.04a	0.06 ± 0.03a	0.109
Nonanal	16.381	0.40 ± 0.02a	0.36 ± 0.01a	0.053	0.43 ± 0.07a	0.24 ± 0.07a	0.068
Naphthalene	18.829	1.47 ± 0.14a	1.05 ± 0.16a	0.066	0.58 ± 0.04a	0.51 ± 0.04a	0.323
Decanal	19.503	0.10 ± 0.01a	0.08 ± 0.01a	0.09	0.07 ± 0.01a	0.06 ± 0.02a	0.923
C	21.818	0.07 ± 0.01a	0.06 ± 0.01a	0.327	0.42 ± 0.04a	0.42 ± 0.05a	0.963
Methyl-naphthalene	22.085	0.29 ± 0.01a	0.22 ± 0.03a	0.075	0.19 ± 0.02a	0.14 ± 0.01a	0.053
D	22.353	0.17 ± 0.03a	0.12 ± 0.02a	0.2	0.18 ± 0.04a	0.09 ± 0.02a	0.078
Tridecanol	22.678	0.09 ± 0.01a	0.1 ± 0.01a	0.114	0.11 ± 0.01a	0.08 ± 0.01a	0.590
β**-Elemene**	23.206	0.16 ± 0.02a	0.18 ± 0.03a	0.62	0.27 ± 0.05a	0.15 ± 0.02b	0.035
Tetradecane	24.934	0.23 ± 0.01a	0.22 ± 0.02a	0.906	0.25 ± 0.03a	0.31 ± 0.04a	0.221
β**-Caryophyllene**	25.494	0.84 ± 0.06a	0.72 ± 0.13a	0.421	1.34 ± 0.12a	0.34 ± 0.17b	0.001
α**-Humulene**	26.416	0.39 ± 0.03a	0.35 ± 0.06a	0.574	0.65 ± 0.06a	0.33 ± 0.04b	0.001
Dibutyl phthalate	37.637	1.70 ± 0.07a	1.51 ± 0.05a	0.053	0.66 ± 0.06a	0.53 ± 0.04a	0.880
E	43.442	0.36 ± 0.03a	0.32 ± 0.03a	0.444	0.34 ± 0.06a	0.25 ± 0.04a	0.234
Squalene	46.037	0.33 ± 0.05a	0.38 ± 0.03a	0.374	0.35 ± 0.05a	0.31 ± 0.05a	0.877

*H_Q_ /H_B_, TYLCV-free tomato plants infested with 3rd-instar nymphs of B. tabaci biotype Q/B; V_Q_ /V_B_, TYLCV-infected tomato plants infested with 3rd-instar nymphs of B. tabaci biotype Q/B. A, Methyl-(1-methylethyl)-Benzene; B, 1-methyl-4-(1-methylethyl)-1,4-Cyclohexadiene; C, [1,1′-Bicyclopentyl]-2-One; D, 1-methyl-4-(1-methylethyl)-2,3-Dioxabicyclo-5-ene; E, 1,2-Benzenedicarboxylic acid, bis (2-ethylhexyl) ester. Values are means ± SE. Means with different letters are significantly different according to the Tukey test at P < 0.05. Significantly different pairs are in bold*.

### Attraction verification of *E. formosa* to synthetic volatiles

**Figure 3:**
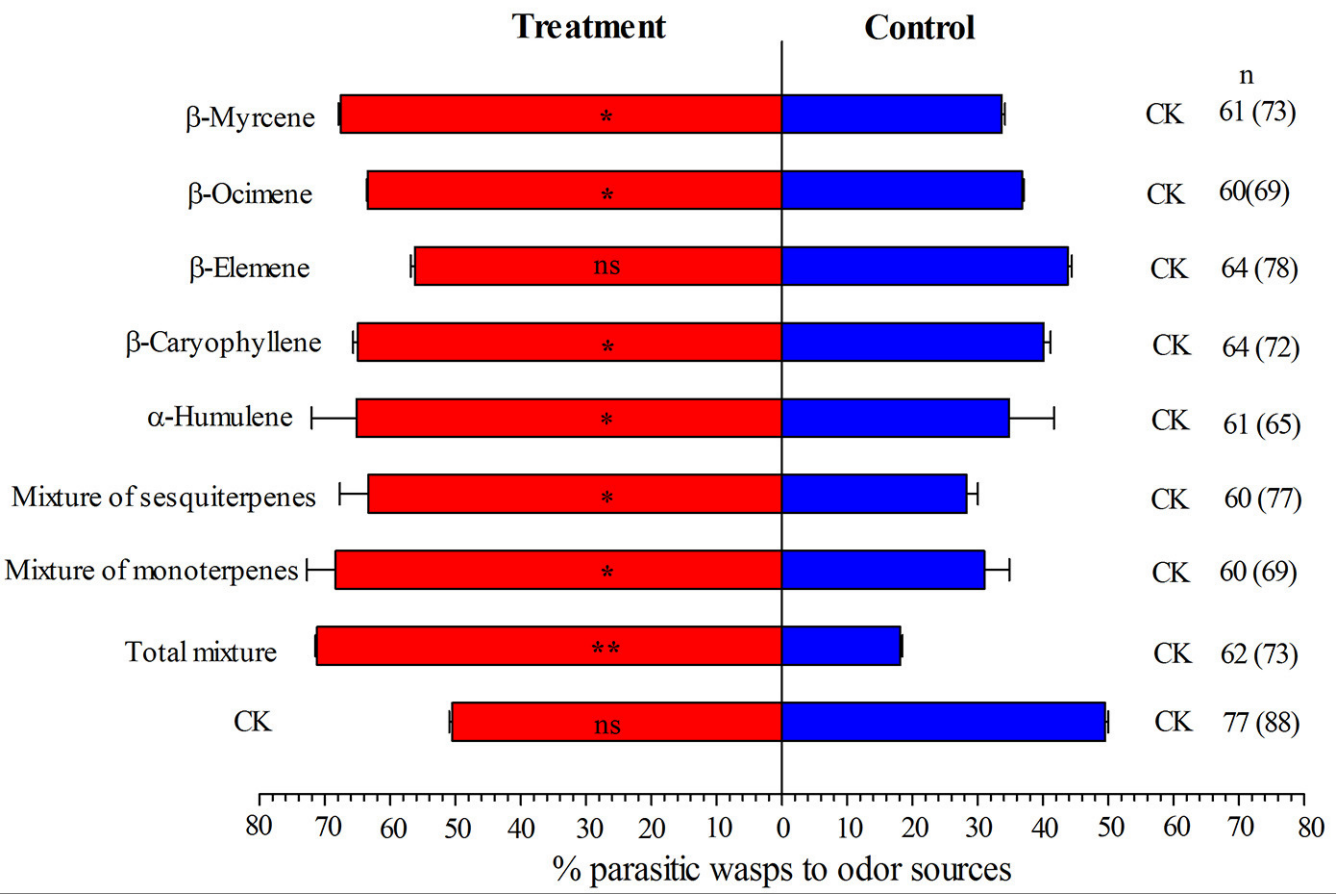
Responses of *Encarsia formosa* to synthetic volatiles and paroline oil (CK) in a Y-tube olfactometer. Bars (means + SE) indicate the percentages of wasps choosing either of the odor sources. The numbers to the right of bars indicate the number of wasps making a choice, and the total number of wasps used in the assay is indicated in parentheses. Asterisks indicate significant differences between the treatment pairs (chi-square test; ^*^P < 0.05; ^**^P < 0.001; ns, not significant).

Results from olfactory test indicated that the mixture of monoterpenes, mixture of sesquiterpenes and mixture of all synthetic volatiles were all strongly attractive for *E. formosa*. In the meanwhile, synthetic β-Myrcene, β-Ocimene, β-Caryophyllene, and α-Humulene attracted *E. formosa* females (Figure 3). β-Elemene did not attract significantly *E. formosa* females (Figure 3).

## Discussion

In addition to the parasitoid *E. formosa*, the current study included two major threats on the tomato plant: biotypes Q or B of *B. tabaci* and the virus TYLCV. In the experiments in which the parasitoid could choose between *B. tabaci* Q and B on tomato leaves in the laboratory and on tomato plants in the greenhouse, the results proved to follow the same trend, i.e., *E. formosa* parasitized significantly more whitefly Q nymphs than B nymphs on TYLCV-infected plants but not on TYLCV-free tomato plants. The results of Y-tube olfactometer assays also showed that naive females of *E. formosa* exhibited a stronger olfactory preference for tomato plants infested with 3rd-instar nymphs of biotype Q than B when the plants were infected with TYLCV but not for the TYLCV-free plants. This would be helpful for the control of whiteflies populations and virus spread in the field, because the parasitoids, the higher trophic level, prefer to parasitize *B. tabaci* Q biotype (the prominent species in China and vectoring TYLCV widely) on TYLCV-infected plants.

Results from other systems also indicated that parasitoid attraction or parasitism rate increased when the herbivore-infested plant was infected by a pathogen (Cardoza et al., 2003; Tack et al., 2012; Ponzio et al., 2016b). For instance, the parasitoid *Cotesia glomerata* was more attracted to *Brassica nigra* plants that were both infested by *Pieris brassicae* larvae and infected by *Xanthononas campestris* than to plants that were only infested with the larvae (Ponzio et al., 2016b). However, other reports indicated that simultaneous attack by an herbivorous insect and a pathogenic fungus did not affect parasitoid foraging (Rostas et al., 2006), indicating that such effect on parasitism foraging may differ with different research systems (Ponzio et al., 2013).

The different responses of *E. formosa* to *B. tabaci* Q vs. B on TYLCV-infected tomato plants vs. TYLCV-free tomato plants was related to quantitative (but not qualitative) differences in certain components of the volatiles emitted by the tomato plants; such differences were not evident in the volatiles emitted from TYLCV-free plants infested with *B. tabaci* Q vs. B. There are also other studies indicated that quantitative differences in volatiles released from plant could alter parasitoid behavior (McCormick et al., 2012; Ponzio et al., 2016b). For example, the olfactometer differences of parasitoids *C. glomerata* on different plant treatments are due to quantitative differences of released volatile compounds of Brussels sprout plants (Ponzio et al., 2016b).

In this present study, the main components of the volatiles that exhibited quantitative changes in response to simultaneous attack by TYLCV and *B. tabaci* were the terpenoids β-Myrcene, β-Ocimene, β-Caryophyllene, α-Humulene, and β-Elemene. The quantities of these components were higher in TYLCV-infected tomato plants, but not TYLCV-free tomato plants, were infested with *B. tabaci* Q rather than B. In subsequent olfactometer assays with synthetic compounds, *E. formosa* was attracted to β-Myrcene, β-Ocimene, β-Caryophyllene, and α-Humulene. β-Myrcene has been proved to strongly attract *E. formosa* adult (Zhang et al., 2013b). Because odors emitted by plants are mixtures of volatile compounds, a complete behavioral response of associated organisms is more likely to be elicited by multiple compounds than by single compounds (Li et al., 2014). Consistent with that view, *E. formosa* was strongly attracted to the mixture of β-Myrcene and β-Ocimene, the mixture of β-Caryophyllene, α-Humulene, and β-Elemene, and the total mixture.

In addition, plants will attempt to defend themselves by activating the salicylic acid (SA), jasmonic acid (JA), and ethylene (ET) signaling pathways, when threatened by herbivores or pathogens (Ponzio et al., 2016b; Su et al., 2016). Feeding by *B. tabaci* B biotype nymphs could induce SA defenses and suppress JA defenses of Arabidopsis (*Arabidopsis thaliana*) plants (Zarate et al., 2007; Zhang et al., 2013a). Previous studies found that challenges by *B. tabaci* Q or B adults have different effects on plant defenses, and that TYLCV infection alters the plant's defense response to *B. tabaci* (Shi et al., 2013, 2014). Moreover, *TPS7* encoding an enzyme that catalyzes the formation of β-Myrcene, *TPS12* encoding an enzyme that catalyzes the formation of the sesquiterpenes β-Caryophyllene and α-Humulene, and *TPS5* (which encodes monoterpenes) are important terpene synthase (TPS) genes in tomato and are induced by JA or SA treatment (Vasiliki Falara et al., 2011; Simon Zebelo et al., 2014). Therefore, it is speculated that feeding by *B. tabaci* Q vs. B nymphs on TYLCV-infected tomato plants may activate different defense singling pathways, altering the expression of SA-regulated and JA-regulated genes that encode volatile terpenes and thereby affecting *E. formosa* attraction and parasitism (Bruce et al., 2008; Zhang et al., 2013a,b; Wu et al., 2017). This possibility needs more additional and deeper studies.

In conclusion, TYLCV-infected plants infested with the *B. tabaci* Q nymphs were significantly more attractive to *E. formosa* than TYLCV-infected plants infested with *B. tabaci* B nymphs. The difference in attraction was associated with increased quantities of β-Myrcene, β-Ocimene, β-Caryophyllene, and α-Humulene in TYLCV-infected plants infested with *B. tabaci* Q nymphs.

## Author contributions

XL designed and performed the experiments, wrote and revised the manuscript. GC conceived the idea, and performed the experiments. SW conceived the idea and reviewed the manuscript. YZ, WX, and QW coordinated and designed the study. All the authors have read and approved the final manuscript.

### Conflict of interest statement

The authors declare that the research was conducted in the absence of any commercial or financial relationships that could be construed as a potential conflict of interest.
